# Multiple Loci Control Variation in Plasticity to Foliar Shade Throughout Development in *Arabidopsis thaliana*

**DOI:** 10.1534/g3.120.401259

**Published:** 2020-09-28

**Authors:** James Ta, Christine Palmer, Marcus Brock, Matthew Rubin, Cynthia Weinig, Julin Maloof, Daniel Runcie

**Affiliations:** * Department of Plant Sciences, University of California, Davis, California; † Department of Plant Biology, University of California, Davis, California; ‡ Department of Botany, University of Wyoming, Laramie, Wyoming; § Donald Danforth Plant Science Center, 975 North Warson Road, St. Louis, Missouri

**Keywords:** nested association mapping, path analysis, QTL mapping, shade avoidance, *Arabidopsis thaliana*

## Abstract

The shade avoidance response is a set of developmental changes exhibited by plants to avoid shading by competitors, and is an important model of adaptive plant plasticity. While the mechanisms of sensing shading by other plants are well-known and appear conserved across plants, less is known about the developmental mechanisms that result in the diverse array of morphological and phenological responses to shading. This is particularly true for traits that appear later in plant development. Here we use a nested association mapping (NAM) population of *Arabidopsis thaliana* to decipher the genetic architecture of the shade avoidance response in late-vegetative and reproductive plants. We focused on four traits: bolting time, rosette size, inflorescence growth rate, and inflorescence size, found plasticity in each trait in response to shade, and detected 17 total QTL; at least one of which is a novel locus not previously identified for shade responses in *Arabidopsis*. Using path analysis, we dissected each colocalizing QTL into direct effects on each trait and indirect effects transmitted through direct effects on earlier developmental traits. Doing this separately for each of the seven NAM populations in each environment, we discovered considerable heterogeneity among the QTL effects across populations, suggesting allelic series at multiple QTL or interactions between QTL and the genetic background or the environment. Our results provide insight into the development and variation in shade avoidance responses in *Arabidopsis*, and emphasize the value of directly modeling the relationships among traits when studying the genetics of complex developmental syndromes.

Because plants are sessile organisms and require light for energy, their ability to monitor and adjust to their light environment is essential to their fitness (Schmid 1992; Gratani 2014). Consequently, plants have photoreceptors to sense changes in the light environment, and have developmental and physiological responses to optimize fitness under non-optimal light conditions (Kami *et al.* 2010). The shade avoidance response (SAR) is a characteristic suite of responses to the proximity of nearby plants in competition for light, and is widely cited as a primary example of adaptive plasticity (Schmitt *et al.* 2003; Keuskamp *et al.* 2010; Bongers *et al.* 2014). Green plant tissues absorb red light and reflect far-red light, so a change in the ratio of red to far-red light, called the red:far-red ratio (R:FR), signals the presence of nearby vegetation and elicits a SAR in receptive plants (Franklin and Whitelam 2005). The SAR is widely cited as an example of adaptive plant plasticity because the morphological and physiological changes are dramatic, and the adaptive benefit has been demonstrated in multiple populations and species (Morgan and Smith 1979; Dudley and Schmitt 1995; Schmitt *et al.* 2003). The SAR also has detrimental effects on yield in crops, and its genetics and management are important targets for optimizing yield (Ballaré *et al.* 1997; Carriedo *et al.* 2016; Wille *et al.* 2017).

Although the SAR is generally triggered by light quality (*i.e.*, R:FR ratio), the specific morphological changes caused by shade differ across tissues and developmental stages, and also depend on the persistence and intensity of the light quality signal (Casal 2012, 2013). Typical SAR characteristics include changes in phenology, physiology and growth resulting in taller plants, but with reduced biomass, which helps a plant escape competition. Phenological changes usually include delayed germination, accelerated flowering, and accelerated seed set. Delaying germination allows a seed to optimize the light environment upon emergence when shading is temporary, while accelerating flowering is generally a strategy for cutting losses and making some seed when shading is persistent (Casal 2012). Elongated and more up-right organs – such as hypocotyls, petioles and stems – are common responses to reduced R:FR, and this response can help plants overtop neighbors and increase light capture (Casal 2012). However, not all organs display elongation, and adult plants often show other responses such as reduced branching and smaller biomass (Casal 2012; Carriedo *et al.* 2016). These contrasting SAR characteristics suggest that distinct mechanisms mediate the SAR across plant development, and recent research suggests that there are separate regulatory pathways for the SAR between the seedling and adult life stages (Nozue *et al.* 2015). Differentiating the genetic mechanisms of the SAR among developmental stages is a central goal, as they remain less understood.

Not only is there variation in shade effects across developmental stages, but variation in the SAR is also observed across different species and among populations within the same species. For instance, the timing of bud outgrowth in response to shade is accelerated in silver birch (*Betula pendula*), delayed in white clover (*Trifolium repens*), and not affected in *Arabidopsis* (Demotes-Mainard *et al.* 2016). Similarly, a population of *Stellaria longipes* from a prairie environment dramatically elongated stems in response to shading, while a population from an alpine environment showed only a slight increase (Alokam *et al.* 2002). These within and among-species differences are thought to be adaptive (Schmitt *et al.* 2003). For example, elongated stems may help *Stellaria* plants outcompete neighboring vegetation in a prairie, but may not be beneficial in areas that lack crowding and overtopping by other plants (*i.e.*, alpine environments) (Alokam *et al.* 2002). Clinal variation in other environmental variables, such as temperature and precipitation, have also been associated with variation in the SAR across *Arabidopsis* populations (Botto 2015). These results suggest that the SAR can evolve and that populations may harbor useful variation for genetically dissecting and manipulating the SAR in different species.

Despite variability in the SAR among species, many genetic mechanisms involved in sensing and responding to shading by other plants appear to be conserved across species. The phytochromes have been established as a mediator of the SAR in *Arabidopsis* (Franklin *et al.* 2003a, b; Franklin and Whitelam 2005), sorghum (Kebrom *et al.* 2006), maize (Sheehan *et al.* 2007), and tomato (Weller *et al.* 2000; Schrager-Lavelle *et al.* 2016). There are also similar genetic and hormonal mechanisms that control axillary bud growth in response to shade for both *Arabidopsis* and crops. For example, shade repression of axillary bud growth is controlled by the transcription regulator *TB1* in sorghum, and its homologs *BRC1* and *BRC2* in *Arabidopsis* (Carriedo *et al.* 2016). The plant hormones auxin, cytokinin, and strigolactone are known to regulate axillary bud growth in *Arabidopsis* and sorghum (Carriedo *et al.* 2016). Auxin-related genes are upregulated in stem transcriptome profiles in tomato in shade conditions (Cagnola *et al.* 2012). Given the extensive genomic resources available in the model species *Arabidopsis*, studies of the SAR in this species can rapidly identify genes and mechanisms that could be useful for controlling the SAR in crops. For instance, insight on phytochrome function from *Arabidopsis* was used to repress the SAR in tobacco (Robson *et al.* 1996) and potato (Boccalandro *et al.* 2003), leading to increased harvest index and tuber yield, respectively.

Extensive variation in the SAR has been reported for *Arabidopsis*. The SAR for hypocotyl elongation and flowering time showed high genetic variation among 157 *Arabidopsis* accessions studied by Botto and Smith (2002). Botto (2015) additionally examined shade effects in 60 genotypes of *Arabidopsis* across 15 different populations and found that the shade plasticity for some reproductive traits was significantly different across populations and was correlated with environmental differences among populations. The genetic basis of variation in several SAR traits in *Arabidopsis*, including hypocotyl length, petiole length, bolting time, and rosette diameter, has been studied by QTL mapping and GWAS (Jiménez-Gómez *et al.* 2010; Coluccio *et al.* 2011; Filiault and Maloof 2012). Studies of natural variation can complement mutation experiments for discovering novel SAR genes. For example, the circadian clock gene *ELF3* was first implicated in the SAR in *Arabidopsis* in a QTL mapping study (Jiménez-Gómez *et al.* 2010; Coluccio *et al.* 2011).

However, previous QTL mapping studies on the SAR in *Arabidopsis* have been limited in several ways. First, only one QTL mapping experiment has studied the SAR in adult plants (Jiménez-Gómez *et al.* 2010). Second, most studies have been done in single biparental populations, which harbor limited genetic diversity. Third, existing studies have mapped QTL for each trait separately, and have not taken into account the associations between traits. This can limit power when multiple traits are correlated, and can be misled by indirect effects transmitted from one trait to another trait due to developmental and physiological relationships between traits. For example, a higher leaf area index indirectly leads to increases in yield due to higher levels of photosynthesis and carbon assimilates for plant growth (Heuvelink *et al.* 2005). Weinig (2000) showed that the light environment modulated elongation in velvetleaf, and this has indirect effects on fecundity through biomass. Fournier-Level *et al.* (2013) revealed that both genetic background and planting location contribute to life history variation, and that planting location affected indirect QTL effect sizes. Accounting for trait relationships in QTL studies can help describe the similarities and differences among the underlying genetics of early and late developmental SARs in this species.

We use a nested association mapping population (NAM) to characterize the genetic architecture of the SAR in *Arabidopsis thaliana* for four traits: bolting days, inflorescence growth, rosette biomass, and inflorescence biomass (Yu *et al.* 2008). Compared to biparental populations, our NAM population has higher genetic diversity, which increases QTL mapping power and detects QTL that are broadly important across populations. Surprisingly, we find that while there is a shade effect, there is little genetic variation in later developmental SAR compared to earlier developmental SAR. However, we do find 17 SAR QTL among 4 traits, and evidence of an allelic series for many of our QTL. Among these, we find QTL on chromosomes 4 and 5 that colocalize for multiple phenotypes, suggesting pleiotropy for later developmental SAR. To determine if these QTL are truly pleiotropic, we estimate the direct and indirect effects of colocalizing QTL on traits throughout developmental time using path analysis. Because shading involves accelerated flowering, which in turn is associated with smaller plant size and biomass, our hypothesis is that QTL effects on later developmental traits (*e.g.*, biomass) should primarily be indirect. We find that trait associations and direct QTL effects on later developmental traits vary across populations and environments. This suggests that pleiotropy depends on both the genetic background and environment. These results highlight the importance of an integrated view of the genotype-phenotype relationship and the need to not only account for genetics and environment, but also phenotype relationships among traits throughout time.

## Materials And Methods

### Plant material

We used two mapping populations to study the genetics of the shade avoidance response in *A. thaliana*: a nested association mapping (NAM) population consisting of seven biparental populations with 1152 total recombinant inbred lines (RILs) (Brock *et al.* 2020), and a diversity panel consisting of ∼100 diverse accessions (Table S1). Col-0 (186AV) was the recurrent parent of all seven NAM populations. Blh-1 (180AV), Bur-0 (172AV), Cvi-0 (166AV), Ita-0 (157AV), Jea (25AV), Oy-0 (224AV), and Sha (236AV) were the alternative parents. F8 generation RILs were created through single-seed descent, selfing, and bulk multiplication. We obtained seeds for each RIL from the Versailles Arabidopsis Stock Center, and the seeds for the diversity panel accessions from Magnus Nordborg.

### Growth conditions

Seeds were stratified for four days at 4° in 0.15% agar solution and then planted in 4x4-potted trays (East Jordan Plastics: EJP804-200) filled with Sungrow Sunshine Mix #1. To improve germination rates, soil surfaces were flattened with a custom tamper before planting seeds. 2 - 3 seeds of the same RIL were planted in the center of each pot. Each pot was thinned to one plant after one week.

Plants were grown in the Controlled Environmental Facilities at UC Davis in five experiments from 05/13 - 08/15. Light was provided by fluorescent light bulbs at 100 *μ*mol photosynthetic active radiation (PAR), and supplemented by LEDs with different red:far-red ratios (R:FR) to simulate sun (R:FR ratio > 1.0) and foliar shade (R:FR ratio ∼0.5) conditions (Franklin and Whitelam 2005). Daylength in both conditions was set to 16h light, 8h dark and the temperature set to 22°. There were 3 shelves (blocks) for each treatment in each experiment, and in total between 4 - 10 replicates of each RIL per treatment grown over all experiments. Photosynthetic active radiation (PAR) and R:FR were checked using a spectrophotometer at the start of each experiment to verify lighting and sun and shade conditions. Because shade affects germination rate, shade-treated plants were germinated in sun conditions (R:FR > 1.0) for approximately one week to ensure comparable germination rates between sun and shade-treated plants.

Trays were watered with 200 - 300 mL Hoagland solution and rotated 3 times per week. For each block, temperature and humidity were measured continuously using HOBO environmental loggers. Plants were sprayed to prevent and treat diseases and pests whenever necessary.

### Traits measurements

Bolting time, inflorescence height, and dry rosette and inflorescence biomass were measured on each plant. Plants were scored 3 times a week for bolting (BD, measured as days after planting). Inflorescence height was measured from the base of the inflorescence to the tip of the main inflorescence, and was measured approximately right after being scored for bolting, and the first and second weeks after bolting. Because not all inflorescence height measurements were taken at the same time, we estimated the growth rate of the main inflorescence (IG) by taking the difference in height between the first and last inflorescence height measurements and dividing by the number of days between the first measurement and the last measurement. Whole rosettes and inflorescences were harvested two weeks after bolting (immediately after the last inflorescence height measurement), dried, and weighed to obtain dry biomass (RB and IB, respectively for dry rosette and inflorescence biomass).

Data were scanned for obviously erroneous data and measurement error, which were excluded from the subsequent statistical analyses.

### Statistical analyses: QTL mapping

Traits were transformed using the Box-Cox procedure and subsequently z-transformed to satisfy the linear model assumptions of normality and constant variance (transformed data in File S1). We estimated shade responses for each line with the follow mixed linear model:

$$P_{ijkl}=SHELF_{i}+TRT_{j}+RIL_{k}+RIL:TRT_{ij}+e_{ijkl}$$ (1)

where *P* is the phenotype, *SHELF* refers to spatial block, *TRT* is light treatment (sun or shade), *RIL* is the genotype (Recombinant Inbred Line), *RIL:TRT* is the genotype-by-environment interaction, and *e* is the error. *SHELF* and *TRT* were modeled as fixed effects, while *RIL* and *RIL:TRT* modeled as random effects. We fit the model as a Bayesian linear mixed model using the brms *R* package (Buerkner 2017). We used the student family of residuals when fitting the Bayesian mixed models to reduce the influence of potential outliers.

We fit models separately for each of the seven populations to estimate the percentage of total phenotypic variance explained (PVE) by genotype main effect (G-PVE) and gene-environment interactions (*i.e.*, GxE-PVE) for each trait. PVE was calculated by dividing the respective random effect variances by the total phenotypic variance (*i.e.*, the sum of the genetic variance, GxE variance, and residual variance). We then reported the average G-PVE and GxE-PVE over all populations for each trait. We also defined the coefficient of genetic variation in plasticity (CV_p) as the standard deviation of GxE for each trait standardized by the absolute value of the population mean plasticity, which is an alternative measure of the amount of genetic variation in plasticity in a population. Plasticity in this case refers to the differences in the genotype means between the simulated sun and shade conditions. We estimated 95% credible intervals for the shelf fixed effects and PVE estimates (Table S2) as the 2.5% and 97.5% quantiles of the samples from its posterior distribution.

We used the posterior means of the line GxE effects as phenotypes for QTL mapping (posterior means in File S2). QTL mapping was run using the GridLMM package (Runcie and Crawford 2019). GridLMM provides the flexibility of joint QTL mapping in multi-parent populations using linear mixed models, and can also prevent proximal contamination of markers, which improves QTL mapping power (Lippert *et al.* 2011). We developed a forward stepwise algorithm using GridLMM functions to fit multiple-QTL models to our data. By adopting a stepwise approach, we gain greater power to detect QTL by controlling for additional QTL elsewhere in the genome. We first generated genotype probabilities for all markers (obtained from Brock *et al.* 2020) using the R/QTL package (Broman *et al.* 2003). We then performed QTL scans for each shade response trait using the Haley-Knott regression approach (Haley and Knott 1992; Broman *et al.* 2003), including a random effect to account for genetic background effects based on genotypes at all markers >10cM from the testing marker. Since nearby markers were highly correlated, we ran QTL scans using a reduced set of 464 markers by iteratively dropping pairs of markers with a correlation >99% (full marker set in File S3). QTL models were run separately for each population. We combined results across the seven populations for joint QTL mapping by summing the log-likelihoods from each population at the testing marker, and then subtracting from this total the sum of log-likelihoods of null models fit to each population. This log-likelihood ratio was compared to a chi-sq distribution with 6 degrees of freedom for hypothesis testing. We generated a *p*-value threshold by permuting genotypes within each biparental population 1000 times (Cheng *et al.* 2010) and used the 95% quantile of the largest -log10(*p*) values per permutation as the entry *p*-value threshold to control the type I error rate at α=0.05.

We estimated uncertainty in QTL positions using the full set of 10,688 markers by calculating 95% confidence intervals for each QTL using an approach modeled on TASSEL’s stepwise regression method (Bradbury *et al.* 2007). Briefly, we determined confidence bounds around each peak marker by sequentially adding a nearby marker to the QTL model at a greater and greater distance to the QTL peak. We defined the confidence interval bounds as the nearest marker positions that resulted in the QTL peak’s *p*-value being ≤α. The only difference in our method relative to TASSEL is that we determined the confidence intervals on just the first confidence interval scan with no subsequent scans. These QTL confidence intervals were then annotated with known shade avoidance genes (combined list of genes from (Nozue *et al.* 2015; Sellaro *et al.* 2017)). Intervals lacking annotated genes are considered to be novel SAR QTL and are likely to contain novel SAR genes.

To show that our pipeline gives results consistent with other methods, we repeated our analysis of the bolting day shade response (BD_SAR) with three other QTL mapping methods: GEMMA’s LMM (Zhou and Stephens 2012), TASSEL (Bradbury *et al.* 2007), and QTL IciMapping (Meng *et al.* 2015). All methods were run across all populations jointly. For GEMMA and TASSEL, we used the full set of 182,314 SNPs; for QTL IciMapping we used the full set of 10,668 markers (both obtained from Brock *et al.* 2020). Genotype, phenotype, covariate, and annotation data used for these methods are in File S4-S10. For GEMMA, we used the default settings to generate the kinship matrix and to run the linear mixed-model. For TASSEL, we used the default settings to run the stepwise algorithm but limited the maximum number of markers in the stepwise model to 10. We used the JICIM method of QTL IciMapping with the default settings and 1,000 permutations. For GEMMA and TASSEL we included population as a covariate. All methods find QTL on chromosomes 4 and 5, but there are differences in other QTL found (Figure S1 and Figure S2 in Supplementary Material R1, and Table S3). These differences might arise due to the statistical method used to find QTL; GridLMM estimates a separate effect of each marker for each population using Haley-Knott regression, while GEMMA and TASSEL use a GWAS approach that treats each SNP as bi-allelic. Overall, while there are discrepancies between the QTL found between methods, we used GridLMM because we were interested in comparing marker effects between populations. GridLMM can also provide an advantageous combination of controlling for population structure, reducing proximal contamination, and increasing QTL mapping power using a stepwise algorithm not found in any other QTL mapping software.

### Statistical analyses: path analysis

We used a QTL-path analysis to assess whether QTL that are shared between traits have separate direct effects on both traits, or if the QTL effect on one trait can be explained as an indirect effect on a trait expressed earlier in development. We built a path model to explain the developmental relationships among traits based on the time of measurement of each trait. We then fit a QTL-path model by performing a QTL scan for each trait starting with all possible paths from other traits included as fixed covariates. Trait order was determined by collection time and developmental timing: BD -> RB -> IG -> IB. The set of paths included for each later trait consisted of both main effects and plasticity effects of all earlier traits.

To create a final QTL-path model, we collected all colocalizing QTL from the QTL scans and built multi-QTL path models separately for the sun and shade conditions using the R package lavaan (Rossel 2012) with the multiple groups analysis (phenotype data used for path analysis in File S11). We then took the difference of QTL effects between environments to estimate the QTL effects of the shade response. QTL effects reported in the path analysis figures thus represent the differences in QTL effects between sun and shade conditions, unless otherwise specified. We used a backward elimination approach to reduce this model to only terms that were significant in either treatment. For each trait, all QTL and previous traits were included in the initial model as predictors. Non-significant terms (*p* > 0.01) in both treatments were removed through an iterative process: the term with the highest *p*-value was sequentially dropped from the model and then the model was re-fitted until all remaining predictor terms were significant (*p* < 0.01) for that trait in either treatment. Model fit was evaluated according to the comparative fit index (CFI), the root mean square error of approximation (RMSEA), and the standardized root mean square residual (SRMR) (Hu and Bentler 1999). We then used mediation analysis in lavaan to estimate direct and indirect effects of QTL. A description of the equations for the QTL-path scans and the path analysis in lavaan, as well as an explanation of how direct and indirect effects are estimated, can be found in File S12.

### Data availability

Scripts and analyses are available at https://github.com/jkhta/sar_qtl. The Bayesian mixed model and QTL mapping pipeline scripts were run on the FARM cluster at UC Davis. File S1 contains the transformed and standardized phenotype data. File S2 contains the posterior means for the genotype and GxE random effects used for QTL mapping. File S3 contains the markers used for the GridLMM analysis. File S4 contains the genotype file used for the GEMMA analysis. File S5 contains the phenotypes (bolting day shade responses) used for the GEMMA analysis. File S6 contains the family covariate used for the GEMMA analysis. File S7 contains the SNP annotation file used for the GEMMA analysis. File S8 contains the genotype file used for the TASSEL analysis. File S9 contains the phenotypes (bolting day shade responses) and family covariate data used for the TASSEL analysis. File S10 contains the phenotype and genotype information used for the QTL IciMapping analysis. File S11 contains the trait data in sun/shade conditions used for path analysis. File S12 has descriptions on the equations used in the QTL-path scans, and the estimation of direct and indirect effects in lavaan. Supplementary Material R1 contains Figures S1-S3. Table S1 contains the names of about 100 natural accessions grown in addition to the NAM population. Table S2 contains the 95% credible intervals for the shelf fixed effects, G-PVE, and GxE-PVE. Table S3 contains the significant markers detected for the bolting day shade response BLUPs using TASSEL. Table S4 contains the summary statistics from the fitted Bayesian mixed models. Table S5 contains the trait effects - estimated from the path models - for the different populations in the sun condition. Table S6 contains the trait effects - estimated from the path models - for the different populations in the shade condition. Table S7 contains the QTL found for the genotype random effects using GridLMM. Supplemental material available at figshare: https://doi.org/10.25387/g3.12108063.

## Results

### Variation in shade responses among populations

To determine the underlying genetics of SAR variation across a broad panel of *Arabidopsis* accessions, we quantified the genetic variation in shade responses of four later-staged developmental traits in a NAM population consisting of 7 biparental populations and a total of 1152 recombinant inbred lines. Plants showed the classic SAR syndrome: compared to sun conditions, plants in simulated shade bolted faster (-0.58 sd decrease), had faster inflorescence growth (0.27 sd increase), and had lower dry rosette biomass (-0.78 sd decrease) and lower dry inflorescence biomass (-0.15 sd decrease) (Table 1).

**Table 1:** Posterior means of the intercept and treatment fixed effects (Plasticity), and the coefficient of genetic variation for plasticity (CV_p = $\sigma_{GxE}/\left|\mu_{Plasticity}\right|$) averaged over all populations for each trait. Values in parentheses next to each posterior mean are the 95% credible intervals for the means. BD, bolting days; IG, inflorescence growth; RB, dry rosette biomass; IB, dry inflorescence biomass

Trait	Intercept	Plasticity	CV_p
BD	0.57 (0.46 - 0.67)	−0.58 (-0.65 - -0.52)	0.12 (0.06 - 0.19)
IG	0.02 (-0.11 - 0.15)	0.27 (0.19 - 0.36)	1.57 (0.21 - 5.71)
RB	0.86 (0.72 - 0.99)	−0.78 (-0.86 - -0.7)	0.15 (0.09 - 0.24)
IB	0.38 (0.26 - 0.51)	−0.15 (-0.23 - -0.07)	15.96 (0.63 - 20.24)

Overall, the variance in shade responses among genotypes was fairly small, with GxE-PVE ranging between 1.27–5.15%, which is an order of magnitude lower than the variances in genetic main effects, which ranged between 13.81–52.04% (Table S4). However, coefficients of genetic variation in plasticity were moderate to large, ranging from 12 to 1596% (Table 1). We also estimated small GxE variances among the diversity panel, with GxE-PVE ranging between 0.39–4.94% (Table S4).

### Additive QTL

To determine the genetic architecture underlying SAR variation, we estimated shade responses for each line for each of the four traits (BD_SAR, RB_SAR, IG_SAR, and IB_SAR) and used these estimates as phenotypes for QTL mapping. We detected 17 SAR QTL across all shade response traits, with 2 - 8 QTL per trait (Table 2). Interestingly, we detect the most QTL for the bolting day shade response (BD_SAR) (8 QTL) and the least for dry inflorescence biomass shade response (IB_SAR) (2 QTL), even though the GxE-PVE for BD (1.27%) is lower than for IB (5.15%). Our QTL mapping results suggest that the genetic architecture underlying the SAR for later developmental shade responses is polygenic.

**Table 2:** Quantitative trait loci (QTL) for the shade responses of each trait. SNP PVE, percent variance explained for the QTL; Left Bound, left marker of the 95% confidence interval; Right Bound, right marker of the 95% confidence interval. # Genes, number of annotated genes found for QTL. BD_SAR, bolting days shade response; IG_SAR, inflorescence growth shade response; IB_SAR, dry inflorescence biomass shade response. RB_SAR, dry rosette biomass shade response

Trait	QTL	SNP PVE	QTL Marker	Chromosome	Left Bound	Right Bound	# Genes
BD_SAR	BD_SAR1_1	4.71	m_1_28847340	1	m_1_28607852	m_1_29478919	3
BD_SAR	BD_SAR1_2	3.16	m_1_29478919	1	m_1_29400200	m_1_29897126	3
BD_SAR	BD_SAR3_1	1.08	m_3_8066460	3	m_3_8040793	m_3_8658987	6
BD_SAR	BD_SAR4_1	10.98	m_4_41028	4	m_4_41028	m_4_527682	2
BD_SAR	BD_SAR4_2	2.00	m_4_9240644	4	m_4_7937660	m_4_9455527	9
BD_SAR	BD_SAR5_1	4.68	m_5_3142427	5	m_5_3062640	m_5_3475211	1
BD_SAR	BD_SAR5_2	1.37	m_5_7484984	5	m_5_7063023	m_5_8277645	3
BD_SAR	BD_SAR5_3	1.36	m_5_25961748	5	m_5_25950815	m_5_26346630	2
IG_SAR	IG_SAR2_1	2.07	m_2_11683361	2	m_2_11607434	m_2_12272151	0
IG_SAR	IG_SAR4_1	1.36	m_4_16640333	4	m_4_16212324	m_4_17289054	8
IG_SAR	IG_SAR5_1	3.72	m_5_4647184	5	m_5_3799350	m_5_5130837	7
IB_SAR	IB_SAR4_1	3.25	m_4_41028	4	m_4_41028	m_4_527682	2
IB_SAR	IB_SAR5_1	3.80	m_5_4110711	5	m_5_3799350	m_5_5018484	7
RB_SAR	RB_SAR4_1	6.32	m_4_41028	4	m_4_41028	m_4_527682	2
RB_SAR	RB_SAR4_2	2.14	m_4_8938713	4	m_4_8504098	m_4_9455527	7
RB_SAR	RB_SAR5_1	3.92	m_5_3142427	5	m_5_3062640	m_5_4251866	6
RB_SAR	RB_SAR5_2	2.07	m_5_25961748	5	m_5_25637221	m_5_26182104	2

Most SAR QTL were found on chromosomes 4 and 5, and four QTL confidence intervals overlapped for multiple traits, suggesting pleiotropy. A region of ≈500,000 bp on the top of chromosome 4 (*SAR4_1*, around 41,028 bp) was associated with BD_SAR, IB_SAR, and RB_SAR, and explained between 3.25–10.98% of the variation in the SAR found in this population (Table 2). A region of ≈1,500,000 bp in the middle of chromosome 4 (*SAR4_2*, around 8,938,713 bp) was associated with the BD_SAR and RB_SAR. A region on the top of chromosome 5 (*SAR5_1*, between 3,000,000 and 5,000,000 bp) was associated with all four shade response traits, and explained between 3.72–4.68% of the variation among traits. A region at the end of chromosome 5 (*SAR5_2*, around 25,961,748 bp) was detected for BD_SAR and RB_SAR, and explained 1.36–2.07% of the variation. Not all SAR QTL were associated with multiple traits: markers m_2_11683361 and m_4_16640333 were detected only for IG_SAR. This suggests that there are both unique and shared aspects of genetic architecture between later developmental traits in the SAR.

### Evidence for allelic series

**Figure 1:**
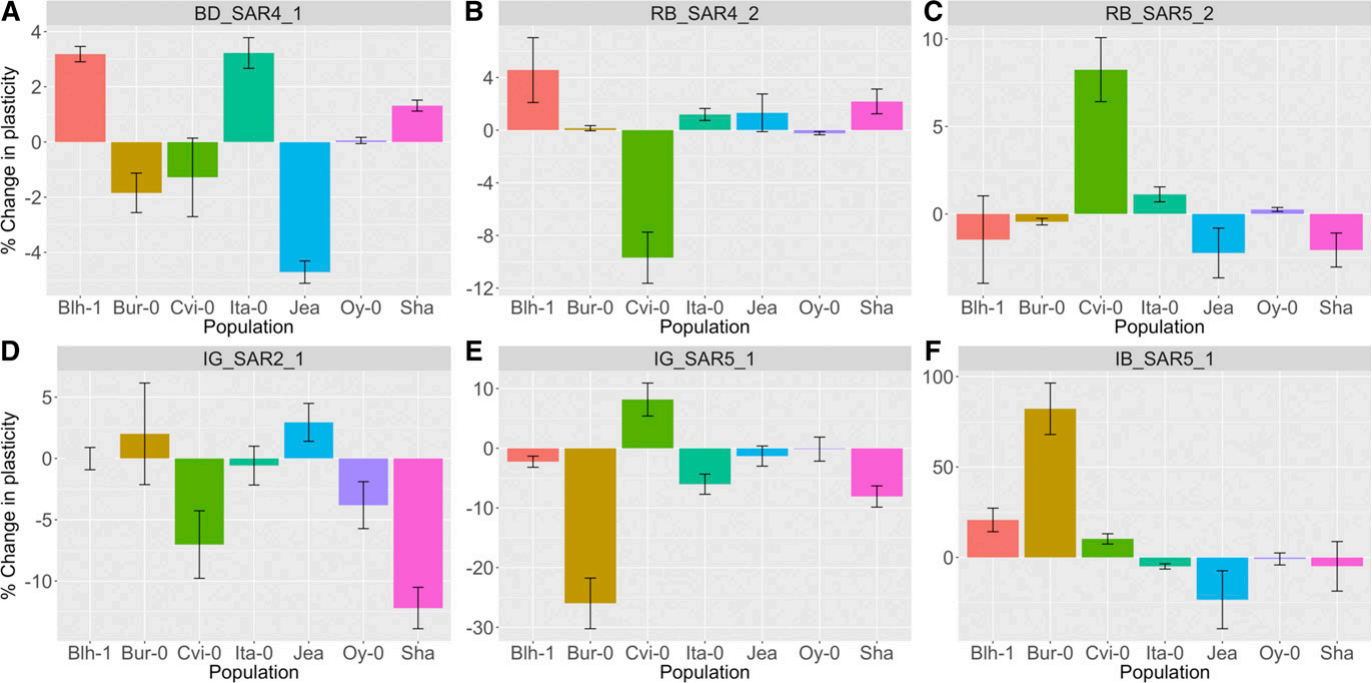
Allelic series among selected SAR QTL. % changes in plasticity relative to the Col-0 allele (allelic-specific change in plasticity / average plasticity) are plotted for selected SAR QTL. Each panel represents a different SAR QTL (panel title). Each bar represents the effect of the non-Col-0 allele in one of the seven different biparental populations. Blh-1, Blh-1 x Col-0; Bur-0, Bur-0 x Col-0; Cvi-0, Cvi-0 x Col-0; Ita-0, Ita-0 x Col-0; Jea, Jea x Col-0; Oy-0, Oy-0 x Col-0; Sha, Sha x Col-0. Error bars represent one standard error of the estimated allele substitution effect.

One of the advantages of a NAM population is that the effect sizes of QTL can be compared across populations. We found clear evidence of multiple functionally distinct alleles at several QTL (Figure 1). For example, at BD_SAR4_1, 3 parents contributed alleles that increased BD_SAR relative to Col-0, 2 contributed alleles that decreased BD_SAR relative to Col-0, and the remaining parents contributed alleles that were similar to Col-0.

At other QTL (*e.g.*, BD_SAR5_1), only one or two parents contributed an allele that differed significantly from the Col-0 common reference (Figure S3 in Supplementary Material R1). We did not observe any QTL where the Col-0 allele was different from every other parent.

### Gene annotation

We annotated each QTL region using a list of genes previously associated with the SAR in *Arabidopsis* from Nozue *et al.* (2015) and Sellaro *et al.* (2017), and listed the number of SAR genes under each QTL (Table 2). Many of these QTL have candidate genes that have been implicated in the mechanism of the SAR through mutant knockouts; however, several have not been shown to vary among natural accessions for the SAR. Additionally, we found 1 SAR QTL that does not contain any previously identified SAR genes: IG_SAR2_1. This region represents a novel SAR QTL, and may provide new insight into the mechanisms of this plasticity.

### Path analysis

**Figure 2:**
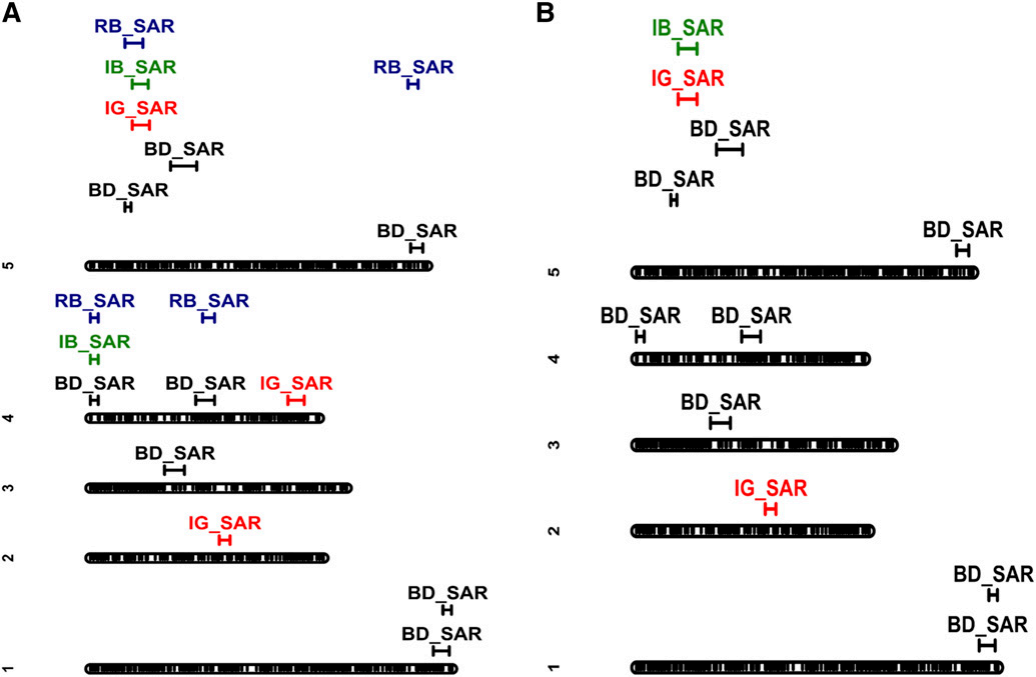
GBS-based single nucleotide polymorphism linkage map of *Arabidopsis* *thaliana*. Roughly 10,668 markers are distributed across 5 chromosomes. (A) Additive quantitative trait loci (QTL) detected without earlier traits as covariates and (B) with earlier traits as covariates. 95% confidence bounds for each QTL are also shown. Overlapping confidence interval bounds suggest colocalization of QTL. BD_SAR, bolting days shade response; RB_SAR, dry rosette biomass shade response; IG_SAR, inflorescence growth shade response; IB_SAR, dry inflorescence biomass shade response.

Next, we used QTL-path analysis to determine if QTL effects on later-staged traits could be explained as indirect effects caused by direct effects of the QTL on earlier traits (in each environment), or earlier shade responses (differences between environments). QTL-path analysis identified a slightly different set of QTL (Figure 2B) as compared to the non-path analysis (Figure 2A). When mapping with earlier traits and shade responses as covariates (Figure 2B), we detected similar QTL on top of chromosomes 4 (for BD_SAR) and chromosome 5 (BD_SAR, IG_SAR, IB_SAR) as compared to the single-trait analyses (Figure 2A). However, the QTL on the top of chromosome 4 is no longer significant for IB_SAR and RB_SAR, and the QTL in the middle of chromosome 4, on the top of chromosome 5, and at the end of chromosome 5 are no longer significant for RB_SAR. These results suggest that these QTL have indirect effects on IB_SAR and RB_SAR.

**Figure 3:**
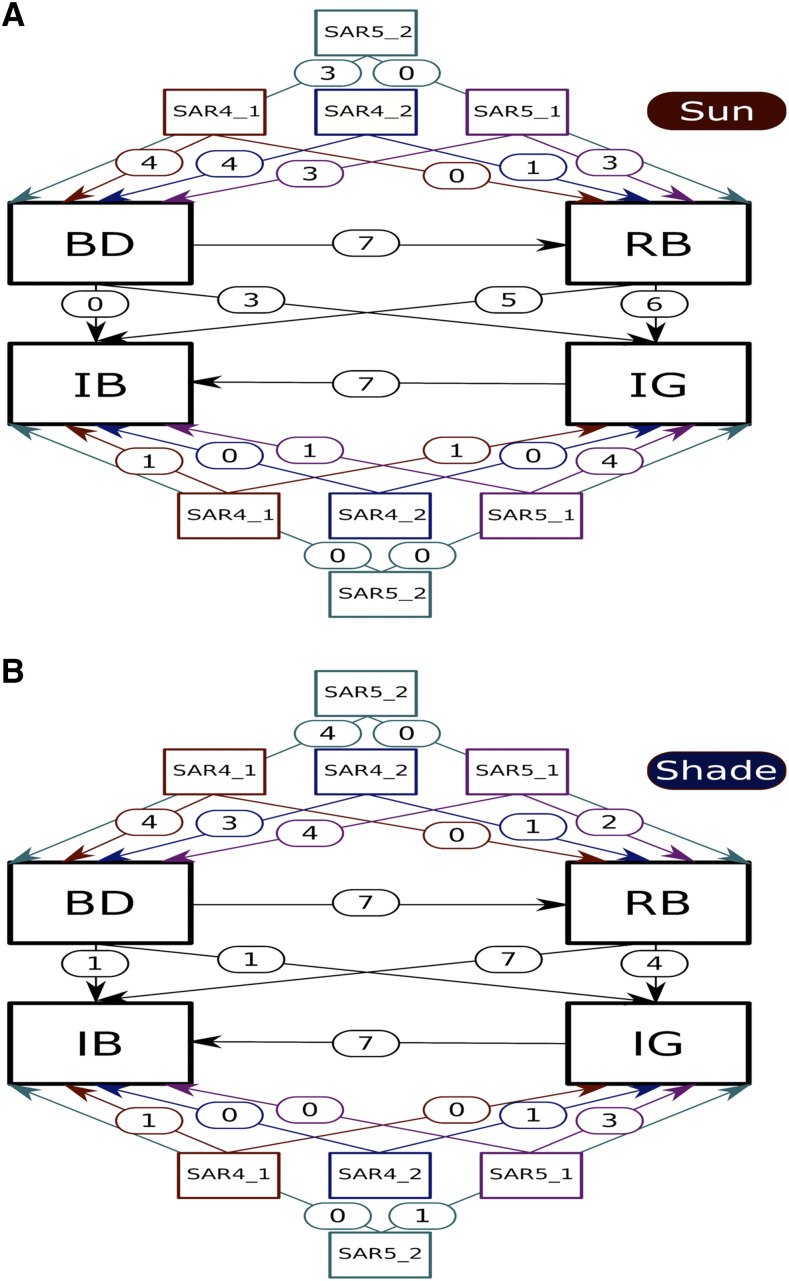
Representation of the fitted path models for sun and shade conditions. Directed arrows represent direct effects. The numbers within the arrows are the number of significant associations across populations (*p* < 0.01). BD, bolting days; IG, inflorescence growth; RB, dry rosette biomass; IB, dry inflorescence biomass. (A) Number of significant path effects in the simulated sun environment. (B) Number of significant path effects in the simulated shade environment. *SAR4_1*, the QTL at the top of chromosome 4 that colocalized for multiple traits. *SAR4_2*, the QTL in the middle of chromosome 4. *SAR5_1*, the QTL at the top of chromosome 5. *SAR5_2*, the QTL at the end of chromosome 5.

To determine if QTL for later-development traits could be explained as indirect effects of colocalized QTL for earlier-development traits, we quantified the direct and indirect effects of each QTL. We modeled QTL effects in sun and shade conditions separately, and then estimated the difference in their effects between sun and shade to determine the effect on shade responses. We then used path analysis to compare the magnitudes of direct and indirect QTL effects among the seven RIL populations (File S12). Fit indices for our models implied adequate fits to the data (average CFI > 0.97, average RMSEA < 0.08, and average SRMR < 0.08 for all models). A conceptual illustration of the path models is shown in Figure 3.

We treated the multiple QTL found on the top of chromosomes 5 for the different shade responses as a single QTL region in our path analysis. This is because the confidence bounds for RB_SAR5_1 overlap with the confidence bounds for BD_SAR5_1, IG_SAR5_1, and IB_SAR5_1.

**Figure 4:**
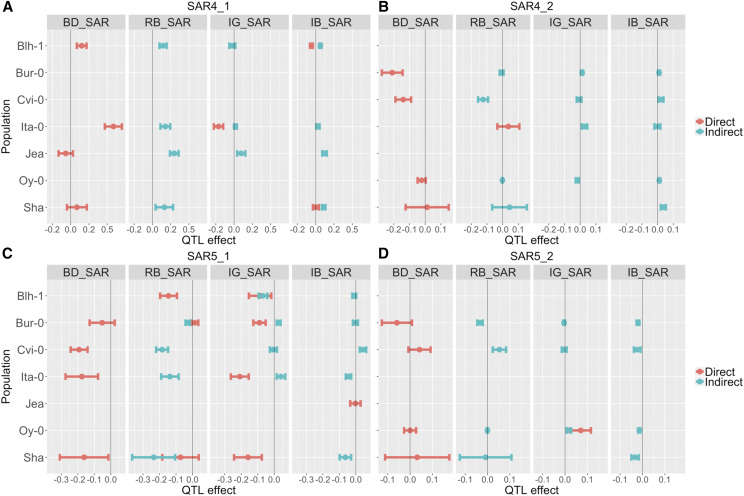
Direct and indirect effects of colocalizing quantitative trait loci (QTL) across populations and traits. Each panel represents a different shade response, going from earlier development (left) to later development (right). The y-axis depicts the different biparental populations, denoted by the non-recurrent parent of the biparental population. Blh-1, Blh-1 x Col-0; Bur-0, Bur-0 x Col-0; Cvi-0, Cvi-0 x Col-0; Ita-0, Ita-0 x Col-0; Jea, Jea x Col-0; Oy-0, Oy-0 x Col-0; Sha, Sha x Col-0. Direct effects are in orange while indirect effects are in teal. Each point represents the estimated QTL effect and the bars represent one standard error of the mean. Non-significant direct effects are not shown; consequently, downstream indirect effects from non-significant direct effects are not shown. BD_SAR, bolting days shade response; RB_SAR, dry rosette biomass shade response; IG_SAR, inflorescence growth shade response; IB_SAR, dry inflorescence biomass shade response. (A) QTL effects for *SAR4_1*, the QTL at the top of chromosome 4 that colocalized for multiple traits. (B) QTL effects for *SAR4_2*, the QTL in the middle of chromosome 4. (C) QTL effects for *SAR5_1*, the QTL at the top of chromosome 5. (D) QTL effects for *SAR5_2*, the QTL at the end of chromosome 5.

Most colocalizing QTL had significant effects in only a subset of the populations (Figure 4). For *SAR4_1*, only populations created with Blh-1, Ita-0, Jea, and Sha showed differences in QTL effects between sun and shade conditions. In the Blh-1 population we observed a positive direct QTL effect on the response to shade for BD_SAR; for the Bur-0 population, however, the direct effect of *SAR4_1* was non-significant. In later developmental traits, indirect effects for *SAR4_1* were non-zero in some, but not all, populations. For example, indirect effects of *SAR4_1* on RB_SAR and IB_SAR were positive in the Blh-1, Ita-0, Jea, and Sha populations.

For *SAR4_2*, we observed direct effects on BD_SAR and RB_SAR, but only indirect effects on IG_SAR and IB_SAR. In contrast, *SAR5_1* shows more direct effects on later developmental traits; including negative direct effects on RB_SAR and IG_SAR in the Blh-1, Ita-0, and Sha populations. Lastly, *SAR5_2* had direct effects on IG_SAR for the Oy-0 population, and indirect effects on RB_SAR and IB_SAR. Interestingly, though we do not detect *SAR5_2* for IG_SAR in our QTL mapping (Figure 2A), we find that *SAR5_2* has direct effects on IG_SAR (Figure 4); this discrepancy might be due to the more stringent significance thresholds in our QTL mapping method compared to our path analysis modeling.

These differences in direct and indirect QTL effects across populations potentially arise due to different trait and QTL effects in different environments. For instance, BD generally had a larger effect on RB in shade conditions than in sun conditions across populations (Table S5 and Table S6). BD effects on RB (RB ∼ BD) ranged between 0.10 - 0.73 in sun and between 0.11 - 0.84 in shade.

### FRI and FLC may underlie the QTL on top of chromosomes 4 and 5

We detected strong QTL on top of chromosomes 4 (*SAR4_1*) and 5 (*SAR5_1*) for multiple shade response traits, including bolting time main effects (averaged over the two environments) (Table S7). These QTL overlap the major flowering repressor genes *FRIGIDA* (*FRI*) and *FLOWERING LOCUS C* (*FLC*), respectively. In low R:FR conditions, flowering is known to be accelerated because the repression of the floral transition by *FRI* and *FLC* is bypassed (Wollenberg *et al.* 2008). Therefore, *FRI* and *FLC* are likely candidate genes for *SAR4_1* and *SAR5_1*.

However, while *FRI* and *FLC* are within *SAR4_1* and *SAR5_1*, respectively, the 95% confidence intervals for these QTL span several Mb, so other loci in these regions may also be involved in these populations. On the other hand, since *FRI* and *FLC* have been extensively studied in *Arabidopsis*, the alleles of these genes have previously been characterized in the majority of the NAM parents in our study. Therefore, if *FRI* and *FLC* are the major causal genes underlying these QTL, the effect sizes and directions across populations should follow predictable patterns.

**Figure 5:**
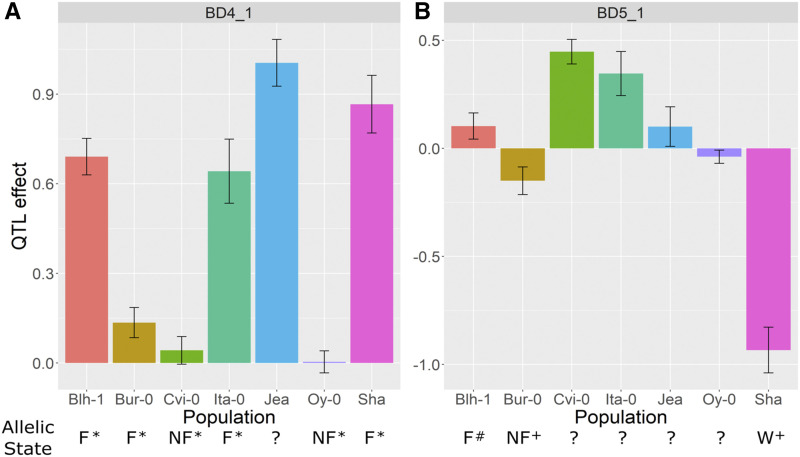
Effects of the BD4_1 and BD5_1 QTL for BD across populations. BD4_1 covers *FRI* and BD5_1 covers *FLC*. QTL effects relative to the Col-0 allele are plotted. Allelic state is either functional (F), non-functional (NF), unknown (?), or weak (W). Symbols next to allelic states represent the references where the information was collected: * (Lovell *et al.* 2013); # (Simon *et al.* 2008); + (Werner *et al.* 2005). Bars represent one standard error of the estimated effect of the non-Col-0 allele.

For instance, Col-0, Cvi-0, and Oy-0 have a non-functional *FRI* allele, while Blh-1, Bur-0, Ita-0, and Sha have a functional *FRI* allele (Lovell *et al.* 2013). Consequently, if *FRI* was the main driver of variation at the BD4_1 QTL, we expect that the QTL effects on BD to be close to zero for the Cvi-0 and Oy-0 alleles, and positive for the Blh-1, Bur-0, Ita-0, and Sha alleles. We find that the Cvi-0 and Oy-0 alleles do not delay bolting (QTL effect close to 0), while the Blh-1, Bur-0, Ita-0, and Sha alleles delay bolting (positive QTL effect) (Figure 5A). These results suggest that variation at *FRI* is the main driver of variation at the BD4_1 QTL.

Similarly, Col-0 and Blh-1 have a functional *FLC* allele while Bur-0 and Sha have either a weak or non-functional *FLC* allele (Gazzani *et al.* 2003; Werner *et al.* 2005; Simon *et al.* 2008). Consequently, we expect QTL effects at BD5_1 on BD to be close to 0 for the Blh-1 allele, and to be negative for the Bur-0, and Sha alleles. We find that the Blh-1 allele only slightly delays bolting (positive QTL effect close to 0), while the Bur-0 and Sha alleles accelerate bolting (negative QTL effect) (Figure 5B). These results then suggest that *FLC* is the main driver of variation at the BD5_2 QTL.

## Discussion

### General findings

We used a nested association mapping (NAM) population to investigate the diversity and genetic basis of variation in developmental responses to shade in *Arabidopsis thaliana*. Our study is the first in *Arabidopsis* to search for quantitative trait loci (QTL) for the shade responses of several late-development traits, including inflorescence growth (IG), rosette biomass (RB), and inflorescence biomass (IB), and includes a much greater sampling of genetic diversity than previous QTL mapping studies of the shade avoidance response (SAR) in this species (Jiménez-Gómez *et al.* 2010; Coluccio *et al.* 2011). Because of the large size of our study, the power of the NAM population, and the assessment of new SAR traits, we find at least one novel QTL that may be useful for future fine-mapping studies to discover genes involved in SAR regulation. Of the 17 SAR QTL we detect, only a few overlap with those found by Jiménez-Gómez *et al.* (2010), who also measured the SAR for later developmental traits. Similar to Jiménez-Gómez *et al.* (2010), we detect QTL near the end of chromosome 5. However, we also detect QTL on chromosome 1, on chromosome 4, and on top of chromosome 5 that were not detected by Jiménez-Gómez *et al.* (2010). This might be due to the greater genetic variation in the NAM population compared to the Bay-0 x Sha population, but the differences in detected QTL might also be due to the differences in the measures used for the shade responses. Jiménez-Gómez *et al.* (2010) used subtraction and residual indices on untransformed data, while we used genotype plasticity estimated from mixed models on transformed data. These discrepencies: the accessions that are represented by the RIL populations, the measure used as the SAR, and the use of untransformed or transformed data, could lead to the contrast in QTL profiles seen between studies. We also use path analysis to determine the mechanisms of pleiotropy among QTL, and discovered that some QTL effects on later development can be explained as effects on earlier development. Fournier-Level *et al.* (2013) also reported increased indirect QTL effects on later developmental traits. However, this depends on the genetic background and environment. Overall, our work describes how foliar shade and genetics influence traits across developmental time.

### Magnitudes of genetic variation in traits and trait plasticity

Our ability to detect QTL depends on the percentage of phenotypic variation that is due to genetic variation, which can be quantified by the percent variance explained (PVE) statistic. We find that variation in the SAR (GxE) among our NAM lines explained very little of the overall variation in any of the traits we measured (GxE-PVE ranged between 1.27–5.15%). This is an order of magnitude lower than the amount of variation explained by genotype main effects (G-PVE ranged between 13.81–52.04%), and also much lower than the amount of residual, or unexplained variation (E-PVE, which ranged between 46.69–81.04%, Table S4). We also observed lower GxE-PVE among our traits in this NAM population compared to the ∼15% GxE-PVE observed for hypocotyl elongation in a panel of 180 *Arabidopsis* accessions (Filiault and Maloof 2012). The low GxE-PVE was not a result of limited diversity among the eight NAM parents, as we observed similar GxE-PVE (< 5% across all traits) in a diversity panel of ∼100 accessions. This suggests lower variation in how *Arabidopsis* accessions respond to shade during later development when compared to the shade response in earlier development. Dechaine *et al.* (2014) also observed higher GxE variation for early internode elongation compared to later internode elongation in *Brassica rapa*, suggesting that decreased GxE variation for later developmental traits is prevalent across multiple species. However, differences in chambers and lighting conditions compared to Filiault and Maloof (2012) could also contribute to differences in GxE-PVE.

However, as a measure of the magnitude of plasticity variation, GxE-PVE can be misleading if the variation attributable to genotype main effects (G-PVE) is large (as this contributes to the total variation independently of GxE). We therefore also report the coefficient of genetic variation in plasticity (CV_p) as a metric of the magnitude of gene-environment interactions. CV_p compares the genetic variation in plasticity to the average plasticity across the populations. By this metric, traits for which some lines respond moderately to shade while others do not may be scored as showing higher genetic variation in plasticity than traits where all lines show strong plasticity, but vary in their magnitude. By the CV_p statistic, we observed considerable variation in the SAR of our traits (CV_p ranged between 12–1596%).

### Genetic diversity at key QTL

By using a multi-parent population, we were able to compare the effects of the same QTL across different donors. Our results provide evidence of allelic series for many of our SAR QTL. Allelic series have previously been observed in *Arabidopsis* for flowering time (Salomé *et al.* 2011) and seed dormancy (Kerdaffrec *et al.* 2016), and allelic variation has also been described for traits in response to shade. McNellis and colleagues (1994) described different allelic classes of *cop1* mutants and their effects on hypocotyl elongation in both simulated canopy shade and end-of-day far-red light treatments. Previous QTL mapping of the SAR in seedlings and adult plants have found two distinct alleles of *ELF3* that regulate hypocotyl elongation and bolting date in response to shade (Jiménez-Gómez *et al.* 2010; Coluccio *et al.* 2011). Similar to Jiménez-Gómez *et al.* (2010), we find different alleles for bolting time in response to shade as well as other later developmental shade responses. In our population, however, we detect more than two functionally distinct alleles for multiple QTL across all of our traits. The alleles vary in effect sizes, from small to moderate, and while most alleles change the plasticity less than |15%|, some alleles change plasticity as much as |80%|. This is similar to the magnitudes of the effects of polymorphisms on the shade response in genes like *COP1* and *ELF3* (McNellis *et al.* 1994; Jiménez-Gómez *et al.* 2010). In comparison, allelic effects on BD main effects were much higher (Table 1 and Figure 5), with alleles changing the average BD by as much as 0.95/0.57 = 163%. Our results suggest that while the range of allelic effects and their effect sizes on the SAR are small-to-moderate, allelic series are still important for variation in the SAR.

An allelic series can be caused by several possible mechanisms. 1) Multiple functionally distinct alleles may be present at the same gene among the 8 NAM parents, such as strong, weak, and non-functional versions of the same gene. 2) The causal variants in each of the 7 NAM families may reside in different genes, but we are unable to resolve multiple QTL due to the limited mapping resolutions within each family (our average QTL width was 0.82 Mb). 3) Even if there are only two functionally distinct alleles at the locus, the average effect of an allele may differ among the NAM families due to differences in genetic background, such as epistatic interactions with variants at other regions of the genome.

Distinguishing among these alternative models will require fine-mapping of each QTL across the NAM families and is beyond the scope of this study. However, our path analysis of the relationships among QTL and traits provides evidence that genetic background effects may be important. We observed several cases of colocalizing QTL among multiple traits, including QTL on chromosomes 4 (*SAR4_1*) and 5 (*SAR5_1* and *SAR5_2*). Using path analysis, we demonstrated that at least some of the QTL on later traits could be explained as indirect effects of the QTL effects on earlier traits during development. However, the breakdown between direct and indirect QTL effects varied among populations and between the sun and shade environments. If the functional relationships among traits vary among populations, then even if a QTL has the same effect on an early developmental trait among populations, the indirect effect of the QTL on a later trait may vary. This would then appear as an allelic series for the later trait. In this study, we only measured four later-development traits. Had we been able to observe many more traits throughout development, we would have been able to further characterize colocalizing QTL to distinguish allelic series of direct effects from allelic series that are the result of different indirect effects through trait relationships.

### FRI and FLC as candidate genes

We found two colocalizing QTL on chromosomes 4 and 5 (*SAR4_1* and *SAR5_1*) for multiple shade responses and provided evidence that *FRI* and *FLC* are the drivers of variation at these loci. *FRI* and *FLC* are flowering repressor genes that control the initiation of flowering, and previous studies have estimated that they are responsible for over 70% of natural variation in flowering time in *Arabidopsis* (Lempe *et al.* 2005; Shindo *et al.* 2005). However, under shade conditions, the effects of *FRI* and *FLC* are bypassed and flowering is accelerated (Wollenberg *et al.* 2008). Because of the association of *FRI* and *FLC* with accelerated flowering in shade, as well as the correlations of flowering time with plant size and inflorescence height (Mitchell-Olds 1996; Gnan *et al.* 2017), it is not surprising that we detect loci that overlap with *FRI* and *FLC* for our traits since our populations carry functionally distinct alleles of both genes (Werner *et al.* 2005; Simon *et al.* 2008; Lovell *et al.* 2013).

However, this logic suggests that *SAR4_1* and *SAR5_1* should only affect the later developmental traits indirectly through its effects on bolting time. But this is not supported by our results. *SAR4_1* and *SAR5_1* have direct effects on rosette biomass and inflorescence growth in some populations, even after correcting for flowering time (Figure 4), indicating that *FRI* and *FLC* directly influence variation in other traits besides flowering. Consistent with these results, Deng *et al.* (2011) showed that *FLC* binds to genes that regulate vegetative development (*e.g.*, *SPL15* and *SPL3*) in addition to genes involved in the floral transition and floral patterning pathways. Similarly, allelic variation in *FRI* has pleiotropic effects on growth rate, flowering time, and water-use efficiency (McKay *et al.* 2003, 2008; Lovell *et al.* 2013). However, another possibility is that the effect of bolting time on later developmental traits is not entirely linear, and our path analysis only accounts for the linear relationship between traits.

### Future work

The SAR is a widely studied example of plant plasticity, and has important implications in plant breeding and agriculture due to its negative effects on yield. Using natural variation to identify important loci for the SAR can help identify genes that both improve our understanding of the mechanisms underlying the SAR and may attenuate the SAR to improve yield in crops. Our study provides insight into the genetic architecture of the SAR in adult plants, and found at least one novel SAR locus. The loci that we describe represent opportunities for future fine-mapping studies to identify new casual variants. Furthermore, several of the previously identified genes located within our other SAR QTL have not been implicated in the natural variation of the SAR and may be worth further study.

Our path analysis results also show a complex, temporal element to the underlying genetic architecture of the SAR, where QTL directly affect earlier – but not later – developmental traits. For instance, *SAR4_2* had direct effects on the shade responses of BD and RB but not IG and IB. An intriguing future direction would be to investigate the temporal dynamics of the SAR development in mature plants. Shade effects on hypocotyl elongation in response to shade are detectable within hours (Cole *et al.* 2011). Our traits were measured over days or weeks so we could not measure short time-scale effects. However, the SAR in adult plants may be amenable to high-throughput phenotyping studies, which could capture genetic changes at hourly (or even finer) time-scales. Numerous studies have used imaging pipelines and time-series data to capture the genetic architecture of plant growth (Zhang *et al.* 2017; Knoch *et al.* 2020), and studies that leverage the same technology to study the genetic architecture of plant plasticity over time are emerging (Honsdorf *et al.* 2014; Marchadier *et al.* 2019). The SAR can thus serve as a system for future high-throughput phenotyping studies to expand our understanding of natural variation in a plastic and adaptive trait throughout time.
